# A Comparison of Local Endolymphatic Sac Decompression, Endolymphatic Mastoid Shunt, and Wide Endolymphatic Sac Decompression in the Treatment of Intractable Meniere's Disease: A Short-Term Follow-Up Investigation

**DOI:** 10.3389/fneur.2022.810352

**Published:** 2022-02-10

**Authors:** Guiliang Zheng, Yupeng Liu, Jingchun He, Shuna Li, Qing Zhang, Maoli Duan, Jun Yang, Yulian Jin

**Affiliations:** ^1^Department of Otorhinolaryngology-Head and Neck Surgery, Xinhua Hospital, Shanghai Jiaotong University School of Medicine, Shanghai, China; ^2^Shanghai Jiaotong University School of Medicine Ear Institute, Shanghai, China; ^3^Shanghai Key Laboratory of Translational Medicine on Ear and Nose Diseases, Shanghai, China; ^4^Ear Nose and Throat Patient Area, Trauma and Reparative Medicine Theme, Karolinska University Hospital, Stockholm, Sweden; ^5^Division of Ear, Nose, and Throat Diseases, Department of Clinical Science, Intervention and Technology, Karolinska Institutet, Stockholm, Sweden

**Keywords:** Meniere's disease, endolymphatic sac surgery, local endolymphatic sac decompression, endolymphatic sac mastoid shunt, wide endolymphatic sac decompression

## Abstract

**Background:**

Meniere's disease (MD) is an inner ear disorder, characterized by recurrent attacks of vertigo, low-frequency sensorineural hearing loss, tinnitus, and aural fullness. Endolymphatic sac surgery is an effective treatment to control vertigo attacks but without causing a hearing loss for intractable MD. However, the methods and effects of endolymphatic sac surgery have been controversial for many years, and the relationship between the vertigo control rates of different endolymphatic sac surgery methods is not well-documented.

**Objectives:**

This study compared the vertigo control rate, hearing outcome, and quality of life (QOL) among different endolymphatic sac surgery, such as local endolymphatic sac decompression (LESD), endolymphatic sac mastoid shunt (ESMS), and wide endolymphatic sac decompression (WESD).

**Materials and Methods:**

We retrospectively analyzed the patients who underwent endolymphatic sac surgery from January 2008 to June 2019. The control rate of vertigo and QOL scores were compared after 2 years of follow-up. The QOL was scored with validation of the MD patient-oriented symptom-severity index (MDPOSI). The pure tone thresholds of all patients at pre- and postoperation were also compared.

**Results:**

In total, 83 MD patients with complete follow-up data were included in the study, i.e., 20 patients with LESD, 28 patients with ESMS, and 35 patients with WESD. Results showed a better vertigo control with WESD than the other groups (70% with LESD, 71.4% with ESMS, and 88.6% with WESD). The QOL was improved after surgery in all groups in which the difference was statistically significant (QOL, preoperative vs. postoperative, 38.2 vs. 10.1 with LESD, 37.8 vs. 9.6 with ESMS, and 37.6 vs. 8.3 with WESD), respectively. After endolymphatic sac surgery, the hearing was well-preserved in the three groups [pure tone averages (PTAs), dB, preoperative vs. postoperative, 41.0 ± 19.3 vs. 40.8 ± 17.9 with LESD, 39.7 ± 16.4 vs. 40.8 ± 18.2 with ESMS, and 38.5 ± 18.7 vs. 36.6 ± 19.5 with WESD].

**Conclusion:**

Wide endolymphatic sac decompression has a higher vertigo control rate, better improvement of QOL, and relatively higher hearing stability or improvement rate after surgery in patients with MD compared with LESD and ESMS.

## Introduction

Meniere's disease (MD) is an inner disorder characterized by recurrent vertigo, fluctuating hearing loss, tinnitus, and aural fullness (1, 2). Although the peak age of presentation of MD is between 40 and 60 years old, it can present at any age (3). Endolymphatic hydrops is accepted as the pathological feature of MD. However, the etiology and detailed pathophysiology of Meniere's attacks remain controversial. Conservative medical management is usually the first-line treatment after diet and lifestyle changes. Although most patients with MD can be controlled in the early stages, the patients in the advanced stage become difficult to control. Due to repeated attacks of vertigo, patients with intractable MD cannot go to work and participate in other social activities.

The primary goal of MD treatments is to reduce vertigo attacks (4). Maintaining or improving hearing function and minimizing disability are also important considerations in the treatment of MD. When conservative treatment cannot control the symptoms, there are a series of more active treatment options, such as endolymphatic sac surgery, vestibular neurectomy, and labyrinthectomy (1, 4, 5). Compared with vestibular neurectomy and labyrinthectomy, endolymphatic sac surgery is the most commonly used surgical procedure with minimal damage to normal structures and a low rate of hearing loss (6), however, endolymphatic sac surgery has been controversial. Although the reported control rate of vertigo in patients with MD ranged from 33 to 94%, it was once considered an ineffective operation (7–11). Despite many controversies, Paparella's paper published in The Lancet in 2008 still considered endolymphatic sac surgery as an early recommended surgical method for patients with intractable MD (7). It is commonly recognized that the efficacy of endolymphatic sac surgery in the treatment of MD is related to the patient's hearing stage. For example, endolymphatic sac surgery is certainly not the first option for stage IV patients because its postoperative vertigo control rate is only 40–46% (4, 8).

Endolymphatic sac surgery has a history of more than 80 years since it was proposed in 1927 by Portmann (12) and it has evolved to the present. It is mainly divided into the endolymphatic shunt and endolymphatic sac decompression. According to different shunt routes, endolymphatic shunt includes endolymphatic sac subarachnoid shunting (ESSS) and endolymphatic sac mastoid shunting (ESMS). ESSS has been gradually replaced by ESMS because of its disadvantages, such as the risk of intracranial infection and the stability of endolymphatic pressure, which is vulnerable to intracranial pressure. In practice, some surgeons perform local endolymphatic sac decompression (LESD), while others do wide endolymphatic sac decompression (WESD) according to their understanding of endolymphatic sac decompression. Therefore, currently, there are three procedures of endolymphatic sac surgery, namely, ESMS, LESD, and WESD. There are many reports on the efficacy of MD through endolymphatic sac surgery, but few investigations on the comparison of these three procedures for vertigo control rate, improvement of (QOL), and hearing outcomes.

We reviewed the relevant data of patients with MD who underwent different endolymphatic sac surgeries in single center to analyze their efficacy of them.

## Materials and Methods

### Patients

This is a retrospective study of adult MD patients treated with different endolymphatic sac surgeries at Shanghai Jiaotong University School of Medicine affiliated Xinhua Hospital, from January 2008 to June 2019. All patients in this study were definite MD as defined by 1995 American Academy of Otolaryngology-Head and Neck Surgery (AAO-HNS): two or more definite spontaneous vertigo attacks lasting for 20 min or longer, at least one occasion hearing loss, tinnitus or fullness of ears, and vertigo caused by other causes were excluded. All patients were treated with diet control, drug treatment (low salt diet, diuretics, oral, or tympanic steroids) for more than 6 months, and their symptoms were still not effectively controlled. Surgeons from the same neuro-otological surgery team performed these surgeries in different periods, ESMS and LESD from January 2008 to September 2016 and WESD from October 2016 to June 2019. The cases of vestibular migraine, bilateral MD, delayed hydrops, and occupying lesions in the cerebellopontine angle were excluded in this study.

Vertigo class for each patient was calculated from the numerical value (x/y)100: x is the average number of vertigo attacks per month during recent 6 months after surgery and y is the average number of vertigo attacks 6 months before treatment. According to the score, the degree of vertigo control was divided into 5 levels: (A: 0, B: 1–40, C: 41–80, D: 80–120, and E: >120) (13).

Before surgery, each patient's stage of MD was determined. Audiometric parameters used for comparison were pure tone averages (PTA) at 500 Hz, 1 kHz, 2 kHz, and 4 kHz and speech discrimination. Preoperative hearing was defined as the poorest hearing level in the 6 months preceding surgery. The staging was performed according to the average hearing threshold (Stage 1: PTA ≤ 25 dBHL; Stage 2: PTA 26–40 dBHL; Stage 3: PTA 41–70 dBHL; and Stage 4: PTA >70 dBHL) (13). More than 10 dB differences in PTA before and after treatment were regarded as better, <10 dB differences as worse, and the others as no change.

The QOL was scored with validation of the MD patient-oriented symptom-severity index (MDPOSI). The MDPOSI consisted of 30 items. All the questions were posed with an ordinal response format (14). All patients received a preoperative questionnaire survey and were followed up for 24 months. Patients who could not use the postoperative questionnaire were followed up by telephone.

All data were statistically analyzed using Sigma Stat 4.0 for Windows. The ANOVA was used to analyze the differences among groups. The Mann-Whitney *U*-test was used for non-normal data. As for degrees of control rate, the Pearson and Spearman tests were used for different variables. The difference was considered to be statistically significant when *p* < 0.05.

### Surgical Procedure

Local endolymphatic sac decompression: With the patient under general anesthesia, a mastoidectomy was performed in a routine fashion. The mastoid was skeletonized to expose the lateral semicircular canal and the posterior semicircular canal and locate the endolymphatic sac at an intersection angle of the rear lower coordinate quadrant by the long axis of the horizontal semicircular canal and the posterior semicircular canal. The bone just on the surface of the endolymphatic sac in the posterior fossa and around the endolymphatic duct behind the posterior semicircular canal was removed.

Endolymphatic sac mastoid shunt: After a mastoidectomy was performed, the endolymphatic sac was located and the bone just on the surface of the endolymphatic sac in the posterior fossa and around the endolymphatic duct behind the posterior semicircular canal was removed same as LESD procedure. A small incision was opened on the endolymphatic sac with a sharp knife, and a small silicone sheet was embedded at the incision to connect the endolymphatic sac with the mastoid cavity.

Wide endolymphatic sac decompression: A wide mastoidectomy was performed in which the posterior cranial fossa, middle cranial fossa, the sigmoid sinus, and the jugular bulb were widely exposed, such as the dura of the middle cranial fossa, the dura of the posterior cranial fossa in front of the sigmoid sinus anterior to the posterior semicircular canal, which was introduced by Paparella et al. (15). The endolymphatic sac and the endolymphatic duct were then completely visible behind the posterior semicircular canal.

## Results

In total, 83 patients had complete follow-up data. The characteristics of these patients are shown in Table 1. There was no significant difference in gender, age, side, and stages among the three groups (*p* > 0.05).

**Table 1:** Patient characteristics in three groups.

	**LESD *n* = 20**	**ESMS *n* = 28**	**WESD *n* = 35**
**Gender**			
Males/females	11/9	16/12	18/17
Age	53.35 ± 10.31	52.64 ± 9.89	51.08 ± 11.92
Side R/L	9/11	13/15	18/17
Stage	1 2 3 4	1 2 3 4	1 2 3 4
Patients (*n*)	3 9 7 1	5 12 11 0	6 14 15 0

*There was no significant difference in gender, age, side, and stages among three groups*.

### Vertigo Control

**Figure 1:**
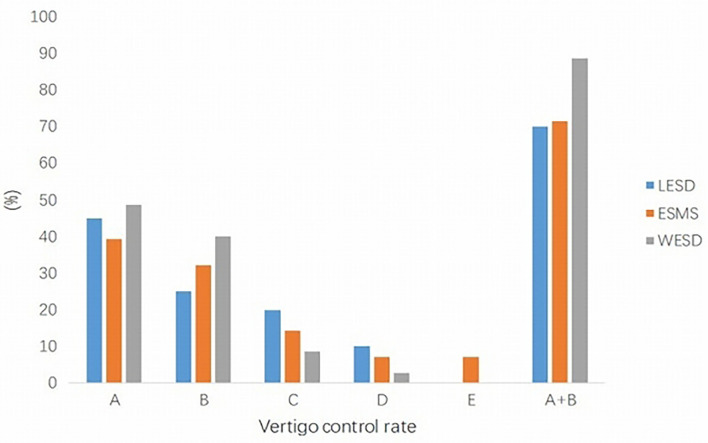
Vertigo control rate in three groups. The satisfactory vertigo control rate (A+B) in the WESD group was better than the other two groups, which were significantly different. WESD, wide endolymphatic sac decompression.

The results of the vertigo control rate are summarized in Figure 1.

For the LESD group, complete control was achieved in 9 patients (45.0%) and substantial control in 5 patients (25.0%). Overall, 14 patients (70.0%) had satisfactory relief from vertigo. No patient was worse than before surgery.

For the ESMS group, complete and substantial control rates were 39.3% (11 patients) and 32.1% (9 patients), respectively. Two patients were worse at 2 months, and one patient received vestibular nerve section 6 months after endolymphatic sac surgery. It was found that the dissected endolymphatic sac was adhered to the surrounding tissue with a lot of granulation and fibrous tissue hyperplasia in this patient.

In the WESD group, 48.6% (17 patients) of the cases showed complete control, whereas 40.0% (14 patients) showed substantial control. Therefore, the satisfactory control rate was 88.6%. There was no patient worse than before surgery.

Complete and substantial relief rates from vertigo were very similar for LESD and ESMS groups (70.0 and 71.4%, respectively). The vertigo control rate in the WESD group was better than the other two groups. When we take satisfactory control of vertigo (complete and substantial) into consideration, there was a statistically significant difference (WESD vs. LESD/ESMS; Figure 1).

### Hearing Outcomes

The audiometric evaluation revealed no significant change in PTA at 24 months follow-up in all three groups. PTA in the LESD group was 41.1 dB preoperatively and 40.8 dB at 24 months evaluation. In the ESMS group, PTA was 39.6 dB preoperatively and 40.8 dB at 24 months after surgery. PTA in the WESD group was 38.5 dB preoperatively and 36.6 dB at 24 months after surgery. PTA improved or stable was in 17 patients and 3 patients with decreased slightly in the LESD group, however, no patient had a hearing loss of more than 15 dB HL. The audiometric evaluation demonstrated that PTA decreased more than 10 dB in 5 patients in the ESMS group at 24 months after surgery, and one of them was decreased significantly with aggravation of vertigo attack. The hearing level was decreased significantly from 37.5 to 67.5 dB 2 months after surgery. The PTA improved or stable was in 30 patients and 5 patients with decreased slightly in the WESD group. However, no patient had a decreased hearing of more than 15 dB HL (Table 2).

**Table 2:** Hearing outcome after endolymphatic sac surgery.

	**LESD**	**ESMS**	**WESD**
Improved	4	4	9
Stable	13	19	21
Decreased	3	5	5

*Patients with improved or stable hearing in LESD, ESMS, and WESD groups were 85.0, 82.1, and 85.7% after 24 months follow-up, respectively (p > 0.05). LESD, local endolymphatic sac decompression; ESMS, endolymphatic sac mastoid shunt; WESD, wide endolymphatic sac decompression*.

### QOL Score

The QOL was scored with validation of the MDPOSI. All patients received a preoperative questionnaire survey and were followed up for 24 months. The patients who could not use the questionnaire survey after surgery were followed up by telephone. MDPOSI scores in the three groups were significantly improved (preoperative vs. postoperative, 38.2 vs. 10.1 with LESD, 37.8 vs. 9.6 with ESMS, and 37.6 vs. 8.3 with WESD). However, there was no significant difference among the three groups (*p* > 0.05).

### Complications

Cerebrospinal fluid (CSF) leakage occurred in 2 patients with WESD procedure intraoperatively. A piece of temporal muscle was used to repair the defect of the dura of the posterior fossa in one patient. For another patient who failed temporal muscle repair, bone wax was used to seal the entrance of the tympanic sinus, and then abdominal fat was taken to fill the mastoid cavity. There was no CSF leakage after surgery.

Another complication was the sigmoid sinus tearing and bleeding, which occurred in 3 patients also with WESD procedure intraoperatively when bone on the surface of the sigmoid sinus was widely removed. Bipolar coagulation or Surgicel was used to stop bleeding.

There was no facial paralysis and posterior semicircular canal injury in all patients with these three endolymphatic sac surgery procedures.

## Discussion

Decision-making of an appropriate surgical procedure for a patient with MD who has failed conservative management is controversial and sometimes difficult. It needs to balance between the control of symptoms and the destructiveness caused by therapeutic modalities. Compared with vestibular nerve section and labyrinthectomy, endolymphatic sac surgery has many advantages, such as less injury and less impact on hearing and vestibular function though relatively low vertigo control (16), which are favorable for patients with MD, especially patients at early or middle stage.

The evolution of endolymphatic sac surgery has experienced a long and tortuous process. As early as 1924, Wittmaack put forward the concept of inner ear hydrops. Guild revealed the key role of the endolymphatic sac in endolymphatic drainage in 1927, which laid an anatomical basis for the establishment of endolymphatic sac surgery. Also in 1927, Portmann, a French doctor, founded the endolymphatic sac opening for the treatment of MD, who achieved certain results and was the first to perform endolymphatic sac surgery for the treatment of MD. Yamakawa et al. (17) modified Portmann's procedure by opening the endolymphatic sac and shunting the endolymphatic fluid to the subarachnoid space in 1954. Twelve years later, Shea made a further modification and developed ESMS. Based on the previous literature (18, 19), the clinical effects of ESMS and ESSS are roughly similar. However, ESSS has been gradually replaced by ESMS because of its distinct disadvantages in intracranial infection and endolymphatic pressure. In the same year of Shea' modification, Shambaugh (20) found that even if the endolymphatic sac was not opened during the surgery, or even without identifying the endolymphatic sac, simply removing the surrounding bone could achieve good results in controlling MD symptoms, Shambaugh therefrom established ESD. An exposure and decompression to the endolymphatic sac is performed in ESD procedure, which does not injure the endolymphatic sac, is more convenient, and has fewer postoperative complications than ESMS. Previous investigations have shown that both EMS and ESD are effective for the treatment of MD, and there is no significant difference in the vertigo control rate between them.

Outcomes of endolymphatic sac surgery have varied from 33 to 94% success for control of vertigo, with most authors reporting success in the range of 70–80% (9, 10, 18, 19). Although endolymphatic sac surgery has been established since 1927, this ambiguity about the effectiveness of surgical intervention in the form of ESD still exists (21–23). Locke (24) reported that the unpredictability of the outcomes of surgical decompression might be related to the difficulty in locating and fully decompressing the endolymphatic sac. Moreover, prolonged exploration of the cystic region may increase the risk of complications due to damage to the cystic cavity and its surrounding structures. Locke recommended that bone should be removed from the superior petrosal sinus to the jugular bulb and from a point medial to the posterior semicircular canal and posterior to the sigmoid sinus (24). The endolymphatic sac was located by measuring the lower limit of the posterior semicircular canal and maintained outside the posterior semicircular canal for decompression in LESD. However, anatomical measurements showed that this was the most variable part. Therefore, decompression limited to this area to reach the endolymphatic sac is unlikely to be reliable (24). Paparella et al. (25) reported that high vertigo control was achieved through wide decompression of the endolymphatic sac and posterior fossa, such as the sigmoid sinus, the jugular bulb, and middle fossa in the study. Gianoli et al. (21) reviewed 35 patients with MD treated by WESD that the complete control rate of vertigo was 85%, and substantial control was 100% in 2 years follow-up. They found that most patients with MD will have an anterior and medial displacement of the sigmoid sinus, and the degree of mastoid pneumatization is generally reduced, reducing the size of the Trautmann triangle and often bringing the sinus in direct contact with the endolymphatic sac. Therefore, extensive decompression, i.e., sigmoid sinus and posterior cranial fossa can better expose the endolymphatic sac and improve endolymphatic drainage. In their study, the vertigo control rate of WESD is higher than that of the LESD. In addition, Ostrowski reported that vertigo control was complete or substantial in 85 and 100% of patients at 1 and 2 years after endolymphatic sac-vein decompression (26).

Although there are many reports on the efficacy of endolymphatic sac surgery in the treatment of MD, few studies compare the effects of ESMS, LESD, and WESD in vertigo control rate and other functional outcomes. ESMS and LESD were performed from January 2008 to September 2016, and WESD from October 2016 to June 2019, respectively, by surgeons from the same neuro-otological surgery team in our single center. The aim of this study was to compare the vertigo control rate, QOL, and hearing outcome among ESMS, LESD, and WESD. Our result showed that the vertigo control rate in LESD and ESMS group was similar (70 vs. 71.3%); however, it was 88% in the WESD group, which was significantly higher than the other two groups.

Another important goal of MD treatment is to maintain or even improve the hearing of the affected ear. Many studies have shown that endolymphatic sac surgery has a slight impact on auditory function (3, 18–21, 24–26). Jameson used round window electrocochleography to measure the electrophysiological changes during ESD and ESS. They found that the low-frequency summating potential (SP) amplitude (500 Hz) had only a small objective change; however, there was no obvious objective change in other measured frequencies or measurement indexes (cm, SP/AP, and CM harmonic distortions). These results suggested that endolymphatic sac surgery has little effect on cochlear electrophysiology (27).

A meta-analysis concluded that endolymphatic sac surgery (sac decompression or mastoid shunt) is effective in controlling vertigo in up to 75% of patients with MD who failed medical treatment, whether short-term or long-term. Once the sac is opened, placing silastic does not add benefit and be deleterious (19). In the present study, the postoperative hearing loss in the ESMS group was more than that in the other two groups, although the difference was not statistically significant. Whether this phenomenon was related to the placement of silicone sheet remains unknown.

Sood et al. (19) reported in a meta-analysis that an average of 72.8% of patients have improved or stable hearing after ESD, and 68% have improved or stable hearing after EMS. In our study, more than 80% of patients were able to maintain stable hearing function after surgery (85.0% with LESD, 82.1% with ESMS, and 85.7% with WESD), which was higher than other reports, which may be related to the fact that all patients were at stages 1–3 preoperatively except one patient at stage 4 (82/83 (98.8%) in which patients at Stage 1+2 accounted for 59%). However, it was reported that ESMS will increase the risk of further hearing loss (19). In the present study, two patients with significant postoperative hearing loss accompanied by aggravated vertigo attack were in the ESMS group in which hearing decreased from 35 to 70 dB HL in one patient who underwent vestibular nerve section *via* retrolabyrinthine approach 6 months later; and from 28.5 to 40 dB HL in another patient 2 months after surgery.

It should be emphasized that preoperative hearing staging is crucial for the selection of surgical procedures for patients with MD. Only patients at the early and middle stages can benefit from endolymphatic sac surgery, such as vertigo control and hearing stability.

Reducing the impact of recurrent MD on daily life and work is also one of the main goals of MD treatment. At present, the main methods to effectively evaluate the impact of MD on patients' life are DHI, MDOQ, and MDPOSI. Murphy et al. (14) reported that there is a highly statistically significant correlation between MDPOSI scores and the impact of MD on patients' life, which can be used to quantify the impact of MD on patients' health status. In our study, MDPOSI was used to evaluate the impact of MD on patients' life before and after surgery. The results suggested that endolymphatic sac surgery could well-reduce the impact of MD on patients and effectively improve patients' QOL because of its relatively higher vertigo control rate and little impact on auditory function. Although there was no significant difference among the three groups, the postoperative score of patients in the WESD group was the lowest, which was consistent with the effective control rate of vertigo.

Regarding time sequence of different surgical procedures, the main reason that we finally preferred WESD as the current procedure of endolymphatic sac surgery is the postoperative vertigo control rate was higher (with a significant difference), relatively higher hearing stability or improvement rate, and better QOL, which is reasonable and suggested by current results. Although there are complications after this procedure, they are relatively few and acceptable. In addition, it was reported that there is a risk of further hearing loss after ESMS (19). Furthermore, during vestibular neurotomy *via* retrolabyrinthine approach in a patient with recurrent MD who underwent ESMS, we found a lot of granulation and fibrous tissue covering the silicone sheet and the incision of the endolymphatic sac, suggesting that the so-called mastoid shunt did not play a role at all.

As for the choice of surgical methods, the main surgical procedures of endolymphatic sac were LESD and ESMS in our center before 2016. The results showed that the vertigo control rate of these two procedures was close, and hearing outcome after ESMS was worse than LESD. The experience of revision endolymphatic sac surgery showed that granulation and fibrous tissue and formation of new bone reoccurred around endolymphatic sac are the main reasons for the failure of endolymphatic sac surgery. Similar situations have been found in our two patients who underwent a second surgery. These results emphasize the importance of wider decompression during endolymphatic sac surgery. Therefore, we carried out WESD since 2016.

The mechanism of the effect of endolymphatic sac surgery is still inconclusive. Gibson (28) suggested that the operation disrupts the vessels of the endolymphatic sac and weakens the activity of the endolymphatic sac, reducing the attack of vertigo. However, Paparella (29) proposed that endolymphatic sac surgery is likely to enhance the blood supply of the sac and improves its absorption function such that alleviates endolymphatic hydrops. A recent study compared the changes of endolymphatic hydrops after different surgeries through enhanced MRI (30). The results showed that endolymphatic hydrops was improved in some patients after ESMS and endolymphatic duct blockage surgery but not in those patients with ESD surgery (30). Taking all these into account, therefore, we suggest that the possible reasons for the better outcome of WESD for intractable MD are as follows: (1) extensive decompression of the endolymphatic sac and dura is supposed to reduce the local impact of granulation tissue, fibrous tissue proliferation, and new bone formation on the endolymphatic sac after surgery; (2) the local hydrostatic pressure and osmotic pressure are changed; (3) extensive decompression affects the blood supply of the endolymphatic sac, resulting in the alteration of the endolymphatic sac activity; and (4) reduced secretion of glycoprotein, in turn, reduced endolymph influx to the endolymphatic sac such that relieve an attack of vertigo.

This study has a few limitations. The first one is that it is a retrospective analysis rather than a randomized controlled prospective study. There may be some deviation in the sophistication of patient selection and imperceptible improvement in surgical skills since the three types of surgical procedures were carried out in different periods though continuous. The second limitation is that these patients with MD in this study were followed up for only 24 months after surgery. We can only draw such a conclusion that functional outcome in the short term among the three procedures was different. However, the outcome in long term needs further research.

## Data Availability Statement

The original contributions presented in the study are included in the article/supplementary material, further inquiries can be directed to the corresponding authors.

## Ethics Statement

Ethical review and approval was not required for the study on human participants in accordance with the local legislation and institutional requirements. The patients/participants provided their written informed consent to participate in this study.

## Author Contributions

JY, MD, and YJ contributed to the study design, critically reviewed, and approved the final manuscript. GZ and YL contributed to the detailed study design and performed data acquisition, statistical analysis, interpretation of results, drafting of the manuscript, and revised the manuscript. JH, SL, and QZ contributed to the methods of statistical analysis and reviewed the manuscript. All authors agree to be accountable for the content of the work, integrity, accuracy of the data, contributed to the article, and approved the submitted version.

## Funding

This work was funded by Cross-Key Projects in Medical and Engineering Fields of Shanghai Jiaotong University (no. ZH2018ZDA11), Clinical Research Cultivation Fund of Xinhua Hospital (nos. 17CSK03 and 18JXO04), and a grant from the Science and Technology Commission Foundation of Shanghai (no. 21S31900600).

## Conflict of Interest

The authors declare that the research was conducted in the absence of any commercial or financial relationships that could be construed as a potential conflict of interest.

## Publisher's Note

All claims expressed in this article are solely those of the authors and do not necessarily represent those of their affiliated organizations, or those of the publisher, the editors and the reviewers. Any product that may be evaluated in this article, or claim that may be made by its manufacturer, is not guaranteed or endorsed by the publisher.
